# Mining clinical relationships from patient narratives

**DOI:** 10.1186/1471-2105-9-S11-S3

**Published:** 2008-11-19

**Authors:** Angus Roberts, Robert Gaizauskas, Mark Hepple, Yikun Guo

**Affiliations:** 1 Department of Computer Science, University of Sheffield, Regent Court, 211 Portobello, Sheffield S1 4DP, UK

## Abstract

**Background:**

The Clinical E-Science Framework (CLEF) project has built a system to extract clinically significant information from the textual component of medical records in order to support clinical research, evidence-based healthcare and genotype-meets-phenotype informatics. One part of this system is the identification of relationships between clinically important entities in the text. Typical approaches to relationship extraction in this domain have used full parses, domain-specific grammars, and large knowledge bases encoding domain knowledge. In other areas of biomedical NLP, statistical machine learning (ML) approaches are now routinely applied to relationship extraction. We report on the novel application of these statistical techniques to the extraction of clinical relationships.

**Results:**

We have designed and implemented an ML-based system for relation extraction, using support vector machines, and trained and tested it on a corpus of oncology narratives hand-annotated with clinically important relationships. Over a class of seven relation types, the system achieves an average F1 score of 72%, only slightly behind an indicative measure of human inter annotator agreement on the same task. We investigate the effectiveness of different features for this task, how extraction performance varies between inter- and intra-sentential relationships, and examine the amount of training data needed to learn various relationships.

**Conclusion:**

We have shown that it is possible to extract important clinical relationships from text, using supervised statistical ML techniques, at levels of accuracy approaching those of human annotators. Given the importance of relation extraction as an enabling technology for text mining and given also the ready adaptability of systems based on our supervised learning approach to other clinical relationship extraction tasks, this result has significance for clinical text mining more generally, though further work to confirm our encouraging results should be carried out on a larger sample of narratives and relationship types.

## Background

Natural Language Processing (NLP) has been widely applied in biomedicine, particularly to improve access to the ever-burgeoning research literature. Increasingly, biomedical researchers need to relate this literature to phenotypic data: both to populations, and to individual clinical subjects. The computer applications used in biomedical research therefore need to support genotype-meets-phenotype informatics and the move towards translational biology. This will undoubtedly include linkage to the information held in individual medical records: in both its structured and unstructured (textual) portions.

The Clinical E-Science Framework (CLEF) project [1] is building a framework for the capture, integration and presentation of this clinical information, for research and evidence-based health care. The project's data resource is a repository of the full clinical records for over 20000 cancer patients from the Royal Marsden Hospital, Europe's largest oncology centre. These records combine structured information, clinical narratives, and free text investigation reports. CLEF uses information extraction (IE) technology to make information from the textual portion of the medical record available for integration with the structured record, and thus available for clinical care and research. The CLEF IE system analyses the textual records to extract entities, events and the relationships between them. These relationships give information that is often not available in the structured record. Why was a drug given? What were the results of a physical examination? What problems were not present? The relationships extracted are considered to be of interest for clinical and research applications downstream of IE, such as querying to support clinical research. The approach taken by the CLEF IE system is one that combines the use of existing terminology resources with supervised Machine Learning (ML) methods. Models of clinical text are trained from human annotated example documents – a gold standard – which can then be applied to unseen texts. The human-created annotations of the gold standard documents capture examples of the specific content that the IE system is required to extract, providing the system with focussed knowledge of the task domain, alongside the broader domain knowledge provided by more general terminology resources. The advantage of this approach is that the system can be adapted to other clinical domains largely through the provision of a suitable gold standard for that domain, for retraining the system, rather than through the creation of new specialised software components or some major exercise in knowledge engineering.

The approach taken to entity extraction in the CLEF IE system has been described in detail elsewhere [2]. This paper focusses instead on relationship extraction in the CLEF IE system. Our approach uses Support Vector Machine (SVM) classifiers to learn these relationships. The classifiers are trained and evaluated using novel data: a gold standard corpus of oncology narratives, hand-annotated with semantic entities and relationships. We describe a range of experiments that were done to aid development of the approach, and to test its applicability to the clinical domain. We train classifiers using a number of different features sets, and investigate their contribution to system performance. These sets include some comparatively simple text-based features, and others based on a linguistic analysis, including some derived from a full syntactic analysis of sentences. Clinically interesting relationships may span several sentences, and so we compare classifiers trained for both intra- and inter-sentential relationships (spanning one or more sentence boundaries). We also examine the influence of training corpus size on performance, as hand annotation of training data is the major expense in supervised machine learning. Finally, we investigate the impact of imperfect entity recognition on relation extraction performance, by comparing relation extraction done over perfect gold-standard entities to that done over imperfect recognised entities. The paper is an expanded version of [3], but extends that paper with a more detailed description of our relation extraction approach, a more thorough discussion of our earlier experimental results, and a report of some additional experiments and their results (specifically those concerning syntactically-derived features and the impact of imperfect entity recognition).

### Previous work

Extracting relations from natural language texts began to attract researchers' attention as a task in its own right during the evolution of information extraction challenges that took place as part of the Message Understanding Conferences (MUCs) (see e.g. [4]), though of course extraction of relational information from text is a part of any attempt to derive meaning representations from text and hence significantly predates MUC. Specifically, relation extraction emerged as a stand-alone task in MUC-7 [5], i.e. requiring participants to extract instances of the employee_of, product_of, and location_of relations, holding between organisations and persons, artefacts and locations respectively, from newswire text. The introduction of this task was part of the factorisation of complex event extraction tasks (for events such as terrorist attacks or joint ventures) that had dominated earlier MUCs, into component tasks that were easier to address and evaluate and would be of relevance in multiple domains (examples of other component tasks factored out in this evolution are named entity recognition and co-reference resolution). The best score obtained on blind test data on this relation extraction task was 75.6% F1-measure (67% precision, 86% recall), where participants had to recognise automatically the entities standing in the relation as well [4]. At the time of MUC-7 the approach adopted by most researchers was to analyse training examples by hand and author patterns to match contexts which expressed the relevant relation. However, even at that time the move away from manually authored extraction patterns towards trainable systems that learned rules or statistical patterns from data was underway, with one participating system (not the highest scoring) using a technique based on automatically augmenting a statistical parser with task specific semantic information obtained from shallow semantic annotation of a training corpus [6].

Since the MUC evaluations there has been increasing work on relation extraction, far more than can be reviewed here. This work can be characterised along several dimensions: the text type (e.g. newswire, scientific papers, clinical reports); the relations addressed (e.g. part-of, located-in, protein-protein interaction); the techniques used (e.g. knowledge-engineering rule-based techniques, supervised learning techniques); whether it was carried out in the context of a shared task challenge for which publicly available task definitions, annotated corpora and evaluation software exist (e.g. the ACE relation extraction challenges [7], the LLL genic interaction extraction challenge [8], the BioCreative-II protein-protein interaction task [9]). We concentrate on the points in this space closest to our own work. There has been little work on relation extraction from clinical texts, presumably because of the difficulty in getting access to texts of this type. In the work carried out to date, extraction of relationships from clinical text is usually carried out as part of a full clinical IE system. Several such systems have been described. They generally use a syntactic parse with domain-specific grammar rules. The Linguistic String project [10] used a full syntactic and clinical sub-language parse to fill template data structures corresponding to medical statements. These were mapped to a database model incorporating medical facts and the relationships between them. MedLEE [11], and more recently BioMedLEE [12] used a semantic lexicon and grammar of domain-specific semantic patterns. The patterns encode the possible relationships between entities, allowing both entities and the relationships between them to be directly matched in the text. Other systems have incorporated large-scale domain-specific knowledge bases. MEDSYNDIKATE[13] employed a rich discourse model of entities and their relationships, built using a dependency parse of texts and a description logic knowledge base re-engineered from existing terminologies. MENELAS[14] also used a full parse, a conceptual representation of the text, and a large scale knowledge base. Note that all these approaches are knowledge-engineering approaches, based on manually authored grammars, lexicons and ontologies. While supervised machine learning has also been applied to clinical text, its use has generally been limited to entity recognition. The Mayo Clinic text analysis system [15], for example, uses a combination of dictionary lookup and a Naïve Bayes classifier to identify entities for information retrieval applications. To the best of our knowledge, statistical methods have not been previously applied to extraction of relationships from clinical text.

By contrast there has been extensive work on relation extraction from biomedical journal papers and abstracts. Much early work in this area and some recent work as well has been done within the hand-written rule base/knowledge engineering paradigm. For example [16-20] all aim to identify gene/protein interactions using simple co-occurrence heuristics or linguistic rules of varying degrees of sophistication to parse sentences and then map syntactic arguments or dependency relations of domain specific verbs into relational structures. Not all the attention has been on protein-protein interactions: [21] discusses such an approach for extracting causal relations between genetic phenomena and diseases and [22] discusses an extension of this approach to a broad range of relations in pharmacogenetics.

In current work on relation extraction more broadly, however, the dominant trend is using supervised ML techniques to train relation classifiers on human annotated texts. Training examples are typically relation instances expressed as a relation type associated with a linked pair of typed entity mentions tagged in a text. The result is a relation classifier capable of recognising relations in entity-tagged text. Approaches differ chie fly according to the ML algorithms and the features employed. Keeping to applications within biomedicine, researchers have explored maximum entropy approaches [23], conditional random fields [24] and rule learning methods such as boosted wrapper induction and RAPIER[25] and inductive logic programming [26]. SVMs have been used for relation extraction, but not extensively in biomedical applications (though see [27]); examples include [28-30]. We use SVMs due to their generally high performance at classification tasks, as it is in these terms that we have recast relation extraction. A wide range of features have been explored for use by supervised ML approaches to relation extraction in biomedical applications. Given a sentence (or text) containing entity mentions whose relationships are to be determined, features investigated have included: orthographic and lexical features of the words between entity mentions and possibly outside the context as well [23,24,27]; part-of-speech and other shallow syntactic features of these words [27]; syntactic information, typically dependency parse information, about the grammatical relations between entity mentions [31]. While all researchers use orthographic and lexical features, the utility of syntactic information remains a topic of debate and one to which the current study contributes.

## Methods

### Relationship schema

The CLEF IE system extracts entities, relationships and modifiers from text. By *entity*, we mean some real-world thing, event or state referred to in the text: the drugs that are mentioned, the tests that were carried out, etc. *Modifiers *are words that qualify an entity in some way, referring e.g. to the laterality of an anatomical locus, or the negation of a condition ("no sign of in ammation"). Entities are connected to each other and to modifiers by *relationships*: e.g. linking a drug entity to the condition entity for which it is indicated, linking an investigation to its results, or a negating phrase to a condition. Note that we treat negation as a modifier word, together with its relationship to a condition. This is in contrast to others (for example [32]), who identify negated diseases and findings as complete expressions.

The entities, modifiers, and relationships are described by both a formal XML schema, and a set of detailed definitions. These were developed by a group of clinical experts, working in collaboration with a computational linguist, through an iterative process, until acceptable agreement was reached. Entity types are manually mapped to types from the Unified Medical Language System (UMLS) semantic network [33], each CLEF entity type being mapped to several UMLS types. Relationship types are those felt necessary to capture the essential clinical dependencies between entities referred to in patient documents, and to support CLEF end user applications. The schema is described further in [34].

**Figure 1:**
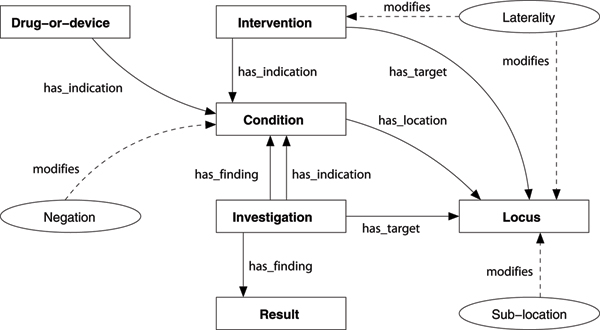
**The relationship schema**. The relationship schema, showing entities (rectangles), modifiers (ovals), and relationships (arrows).

Each relationship type is constrained to hold only between pairs of specific entity types, e.g. the has_location relation can hold only between a Condition and a Locus. Some relationships can hold between multiple type pairs. The full set of relationships and their argument types are shown in Table 1, with a description and examples of each. The schema is shown graphically in Figure 1.

**Table 1:** Relationship types and examples.

**Relation type**	**First argument type**	**Second argument type**	**Description**	**Examples**
**has_target**	**Investigation Intervention**	**Locus**	Relates an intervention or an investigation to the bodily locus at which it is targeted.	• This patient has had a [arg2] lymph node [arg1] biopsy • ... he does need a [arg2] groin [arg1] dissection
**has_finding**	**Investigation**	**Condition Result**	Relates a condition to an investigation that demonstrated its presence, or a result to the investigation that produced that result.	• This patient has had a lymph node [arg1] biopsy which shows [arg2] melanoma • Although his [arg1] PET scan is [arg2] normal ...
**has_indication**	**Drug or device Investigation Intervention**	**Condition**	Relates a condition to a drug, intervention, or investigation that is targeted at that condition.	• Her facial [arg2] pain was initially relieved by [arg1] co-codamol
**has_location**	**Condition**	**Locus**	Relationship between a condition and a locus: describes the bodily location of a specific condition.	• ... a biopsy which shows [arg1] melanoma in his right [arg2] groin • Her [arg2] facial[arg1] pain was initially relieved by co-codamol
**negation_modifies**	**Negation sig nal**	**Condition**	Relates a condition to its negation or uncertainty about it.	• There was [arg1] no evidence of extra pelvic [arg2] secondaries
**laterality_modifies**	**Laterality signal**	**Locus Intervention**	Relates a bodily locus or intervention to its sidedness: *right*, *left*, *bilateral*.	• ... on his [arg1] right[arg2] second toe • [arg1] right[arg2] thoracotomy
**sub_location_modifies**	**Sub-location signal**	**Locus**	Relates a bodily locus to other information about the location: *upper*, *lower*, *extra*, etc.	• [arg1] extra[arg2] pelvic

Relationship types, their argument type constraints, a description and examples. Each example shows a single relation of the given type. Arguments are underlined and preceded by their argument number.  (Adapted from the CLEF Annotation Guidelines, )

Some of the relationships considered important by the clinical experts were not obvious without domain knowledge. For example, in

*He is suffering from nausea and severe headaches. Dolasteron was prescribed*.

domain knowledge is needed to identify the has_indication relation between the drug "Dolasteron" and the "nausea" condition. As in this example, many such relationships are inter-sentential.

A single real-world entity may be referred to several times in the same text. Each of these co-referring expressions is a *mention *of the entity. The schema includes encoding of co-reference between different textual mentions of the same entity. For the work reported in this paper, however, co-reference is ignored, and each entity mention is treated as a different entity. Relationships between entities can be considered, by extension, as relationships between the single mentions of those entities. We return to this issue below.

### Gold standard corpus

The schema and definitions were used to hand-annotate the entities and relationships in oncology narratives, to provide a gold standard for system training and evaluation. By "narrative" we mean letters, notes, and summaries written by the oncologist, describing the patient's care. Most are very loosely structured, and may be described as consisting of general language with a high terminology content, rather than consisting of formulaic sublanguage or boilerplate. Approval to use this corpus for research purposes within CLEF was obtained from the Thames Valley Multi-centre Research Ethics Committee (MREC). The corpus comprises 77 narratives, which were carefully selected and annotated according to a best practice methodology, as described in [34]. Narratives were selected by randomised and stratified sampling from a larger population of 565 000 documents, along various axes such as purpose of narrative and neoplasm. Narratives were annotated by two independent, clinically trained, annotators, and then a consensus annotation created by a third. We refer to the corpus as C77. Corpora of this small size are not unusual in supervised machine learning, and reflect the expense of hand annotation.

Annotators were asked to first mark the mentions of entities and modifiers, and then to consider each in turn, deciding if it had relationships with mentions of other entities. Although the annotators marked co-reference between mentions of the same entity, they were asked to ignore this for relationship annotation. Both the annotation tool and the annotation guidelines enforced the creation of relationships between mentions, not entities. The gold standard is thus analogous to the style of relationship extraction reported here, with relations being assigned between entity mentions, ignoring co-reference. Annotators were further told that relationships could span multiple sentences, and that it was acceptable to use clinical knowledge to infer when a relationship existed. Counts of all relationships annotated in C77 are shown in Table 2, sub-divided by the number of sentence boundaries spanned.

**Table 2:** Relationship counts in the gold standard.

	**Sentence boundaries between arguments**										
	
	**0**	**1**	**2**	**3**	**4**	**5**	**6**	**7**	**8**	**9**	*>***9**
**has_finding**	265	46	25	7	5	4	3	2	2	2	0
**has_indication**	139	85	35	32	14	11	6	4	5	5	12
**has_location**	360	4	1	1	1	1	1	0	0	0	4
**has_target**	122	14	4	2	2	4	3	1	0	1	0
**laterality_modifies**	128	0	0	0	0	0	0	0	0	0	0
**negation_modifies**	100	1	0	0	0	0	0	0	0	0	0
**sub_location_modifies**	76	0	0	0	0	0	0	0	0	0	0
**Total**	1190	150	65	42	22	20	13	7	7	8	16
**Cumulative total**	1190	1340	1405	1447	1469	1489	1502	1509	1516	1524	1540

Count of relations in 77 gold standard documents, sub-divided by the number of sentence boundaries between relations.

### Relationship extraction

**Figure 2:**
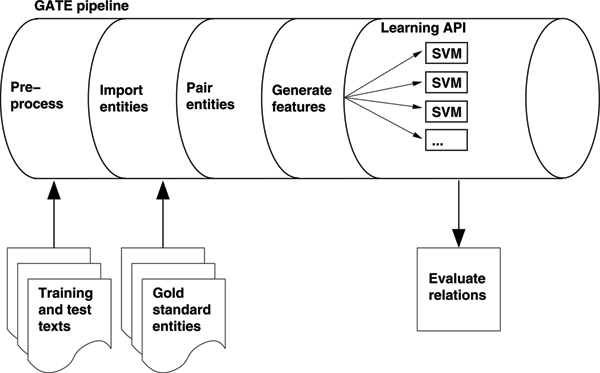
**The relationship extraction system**. The relationship extraction system, as a GATE pipeline.

Our system is built using the GATE NLP toolkit, which is an architecture allowing language processing applications to be constructed as a pipeline of processing components [35]. Documents are passed down this pipeline, being analysed by each component in turn, with the results of this analysis being available to later components. The system is shown in Figure 2, and is described below.

Narratives are first pre-processed using standard GATE modules. Narratives were tokenised, sentences found with a regular expression-based sentence splitter, part-of-speech (POS) tagged, and morphological roots found for word tokens. Each token was also labelled with a more generic POS tag, consisting of the first two characters of the full POS tag. This takes advantage of the Penn Treebank tagset used by GATE's POS tagger, in which related POS tags share the first two characters. For example, all six verb POS tags start with the letters "VB". We will refer to this as a "generalised" POS tag.

After pre-processing, mentions of entities within the text are annotated. In the experiments reported, unless otherwise stated, we assume perfect entity recognition, as given by the entities in the human annotated gold standard described above. Our results are therefore higher than would be expected in a system with automatic entity recognition. It is useful and usual to fix entity recognition in this way, to allow tuning specific to relationship extraction, and to allow the isolation of relation-specific problems. Ultimately, however, relation extraction does depend on the quality of entity recognition. To illustrate this, we provide a comparison with relations learned from automatic entity recognition, in the Results section.

#### Classification

We treat clinical relationship extraction as a classification task, training classifiers to assign a relationship type to an *entity pair*. An entity pair is a pairing of entities that may or may not be the arguments of a relation. For a given document, we create all possible entity pairs within two constraints. First, entities that are paired must be within *n *sentences of each other. For all of the work reported here, unless stated, *n *≤ 1 (crossing 0 or 1 sentence boundaries). Second, we constrain the entity pairs created by argument type [36]. For example, there is little point in creating an entity pair between a Drug or device entity and a Result entity, as no relationships exist between entities of these types, as specified by the schema. Entity pairing is carried out by a GATE component developed specifically for clinical relationship extraction. In addition to pairing entities according to the above constraints, this component also assigns features to each pair that characterise its lexical and syntactic qualities (described further in the following section). The classifier training and test instances consist of entity pairs. For training, an entity pair which corresponds to the arguments of a relationship present in the gold standard is assigned that relationship type as its class – or the class null if there is no corresponding gold standard relation. The classifier builds a model of these entity pair training instances, from their features. In classifier application, entity pairs are created from unseen text, under the above constraints. The classifier assigns one of our seven relationship types, or null, to each entity pair.

We use SVMs as trainable classifiers, as these have proved to be robust and efficient for a range of NLP tasks, including relation extraction. We use an SVM implementation developed within our own group, and provided as part of the GATE toolkit. This is a variant on the original SVM algorithm, SVM with uneven margins, in which classification may be biased towards positive training examples. This is particularly suited to NLP applications, in which positive training examples are often rare. Full details of the classifier are given in [37]. We used the implementation "out of the box", with default parameters as determined in experiments with other data sets.

The SVM with uneven margins algorithm is a binary classifier. Thus to apply it to a multi-class problem requires mapping the problem to a number of binary classification problems. There are several ways in which a multi-class problem can be recast as binary problems. The commonest are *one-against-one *in which one classifier is trained for every possible pair of classes, and *one-against-all *in which a classifier is trained for a binary decision between each class and all other classes, including null, combined. We have carried out extensive experiments (not reported here), with these two strategies, and have found little difference between them for our data. We have chosen to use one-against-all, as it needs fewer classifiers (for an *n *class problem, it needs *n *classifiers, as opposed to *n*(*n *- 1) = 2 for one-against-one).

The resultant class assignments by multiple binary classifiers must be post-processed to deal with ambiguity. In application to unseen text, it is possible that several classifiers assign different classes to an entity pair (test instance). To disambiguate these cases, the output of each one-against-all classifier is transformed into a probability, and the class with the highest probability is assigned. Re-casting the multi-class relation problem as a number of binary problems, and post-processing to resolve ambiguities, is handled by the GATE Learning API.

#### Features for classification

The SVM classification model is built from lexical and syntactic features assigned to tokens and entity pairs prior to classification. We use features developed in part from those described in [29] and [38]. These features are split into 15 sets, as described in Table 3.

**Table 3:** Feature sets for learning.

**Feature set**	**Size**	**Description**
tokN	8*N*	Surface string and POS of tokens surrounding the arguments, windowed -*N *to +*N*, *N *= 6 by default
gentokN	8*N*	Root and generalised POS of tokens surrounding the argument entities, windowed *N *to +*N*, *N *= 6 by default
atype	1	Concatenated semantic type of arguments, in arg1-arg2 order
dir	1	Direction: linear text order of the arguments (is arg1 before arg2, or vice versa?)
dist	2	Distance: absolute number of sentence and paragraph boundaries between arguments
str	14	Surface string features based on Zhou et al [29], see text for full description
pos	14	POS features, as above
root	14	Root features, as above
genpos	14	Generalised POS features, as above
inter	11	Intervening mentions: numbers and types of intervening entity mentions between arguments
event	5	Events: are any of the arguments, or intevening entities, events?
allgen	96	All above features in root and generalised POS forms, i.e. gen-tok6+atype+dir+dist+root+genpos+inter+event
notok	48	All above except tokN features, others in string and POS forms, i.e. atype+dir+dist+str+pos+inter+event
dep	16	Features based on a syntactic dependency path.
syndist	2	The distance between the two arguments, along a token path and along a syntactic dependency path.

Feature sets used for learning relationships. The table is split into non-syntactic features, combined non-syntactic features, and syntactic features. The size of a set is the number of features in that set.

The tokN features are POS and surface string taken from a window of *N *tokens on each side of both paired entities. For *N *= 6, this gives 48 features. The rationale behind these simple features is that there is useful information in the words surrounding the two mentions, that helps determine any relationship between them. The gentokN features generalise tokN to use morphological root and generalised POS. The str features are a set of 14 surface string features, encoding the full surface strings of both entity mentions, their heads, their heads combined, the surface strings of the first, last and other tokens between the mentions, and of the two tokens immediately before and after the leftmost and rightmost mentions respectively. The pos, root, and genpos feature sets are similarly constructed from the POS tags, roots, and generalised POS tags of the entity mentions and their surrounding tokens. These four feature sets differ from tokN and gentokN, in that they provide more fine-grained information about the position of features relative to the paired entity mentions.

For the event feature set, entities were divided into events (Investigation and Intervention) and non-events (all others). Features record whether an entity pair consists of two events, two non-events, or one of each, and whether there are any intervening events or non-events. This feature set gives similar information to atype (semantic types of arguments) and inter (intervening entities), but at a coarser level of typing. The feature sets allgen and notok are combinations of the above feature sets, as specified by the descriptions in Table 3.

For the final two feature sets shown in Table 3, we used the Stanford Parser [39] to parse the C77 corpus. This parser generates a dependency analysis, consisting of a graph of syntactic relations amongst sentence tokens. The feature set dep consists of 16 features derived from the parse, which are only computed when the entities appear in the same sentence (and otherwise take value null). The features encode characteristics of the dependency path connecting the paired entities, of the immediate left context in the dependency analysis of the leftmost entity, and of the corresponding right context of the rightmost entity. The syndist set adds two further features, which firstly count the number of links on the dependency path connecting the paired entities and the number of tokens between the two entities, and then maps these values to labels NEAR, MIDDLE and FAR, to reduce data sparseness.

### Evaluation methodology

We use the standard evaluation metrics of Recall and Precision, which are defined in terms of true positive (TP), false positive (FP) and false negative (FN) matches between relations recorded in a system annotated *response *document and a gold standard *key *document. A response relation is a true positive if a relation of the same type, and with the exact same arguments, exists in the key. Corresponding definitions apply for false positive and false negative. Counts of these matches are used to calculate Recall (*R*) and Precision (*P*) scores, as defined below. The harmonic mean of these two values provides a single combined indicator of performance. This metric, known as *F*1, as also defined below.

$$\begin{array}{ccc} R=\frac{TP}{TP+FN} & P=\frac{TP}{TP+FP} & F1=\frac{2PR}{P+R} \end{array}$$

We used a standard ten-fold cross validation methodology in our experiments. Various tables given later report the results of these experiments, showing recognition scores for the different relation types and for relation recognition overall. The scores for individual relations are produced by computing the *P*, *R *and *F*1 scores for each relation type on each fold, and then macro-averaging these values (i.e. computing their simple mean) across the folds to give the corresponding relation-specific cross-validated score. This approach can produce results that may at first sight seem anomalous, e.g. cases where the *F*1 score for a given relation does not fall between the *P *and *R *scores. Overall scores for relation recognition are produced by first micro-averaging scores for the different relation types within the fold, i.e. simply adding their counts for true-positives, false-negatives and false-positives, and using these summed values to compute *P *and *R *values directly. The resulting combined scores are then macro-averaged across folds to produce the cross-validated overall scores.

The metrics do not say how hard relationship extraction is. We therefore also provide Inter Annotator Agreement (IAA) scores from the creation of the gold standard. The IAA measures the level of agreement between the two annotators who independently annotated each text to produce its double annotation. It is equivalent to scoring one annotator against the other using the *F*1 metric (i.e. treating one annotation as key and the other as response).

IAA scores are not directly comparable here to system extraction scores, as relationship annotation is a slightly different task for the human annotators. The relationship extraction system is given entities, and finds relationships between them. Human annotators must find both the entities and the relationships. Where one human annotator fails to find a particular entity, they can never find its relationships. The raw IAA score does not take this into account: if an annotator fails to find an entity, they will also be penalised for all relationships with that entity. We therefore give a Corrected IAA (CIAA) in which annotators are only compared on those relations for which they have both found the entities involved. In our results, we give both IAA and CIAA, for each relation type and for relations overall. As our results will show, it is clear that it is difficult for annotators to reach agreement on relationships, some more so than others. Further, lower values for IAA than for CIAA show this difficulty is compounded massively by lack of agreement on entities. The level of agreement that is achieved between annotators is often seen as providing an *upper bound *for what can be expected of system performance. The situation here however is complicated by the fact that the gold standard used in training and evaluation is produced by a further consensus process, so that gold standard annotations may exhibit a greater degree of regularity, reliability and correctness than can be expected of the output of any one annotator, making it at least possible for the system to score higher on some relation than the observed annotator agreement level.

A second basis for evaluating system performance is comparison against *baseline *scores for the given task, which are scores that can be achieved using some quite simplistic method. Baseline scores can be viewed as providing a (reasonable) *lower bound *for performance, and the improvement over the baseline is a measure of the benefit achieved by using a more complex approach. For classification tasks, a common baseline is to assign to all members of a group of instances the most common class found for that group within the gold standard. A baseline method for relation extraction will begin with the set of possible entity pairs for each document, as discussed earlier for our relation recognition method proper, where the possible entity pairs are restricted to only those whose entities are of suitable types, and which occur in the same or adjacent sentences, and each entity pair assigned as their class either a relation type from the gold standard or the value null. An obvious baseline approach is to subdivide this overall set of instances (i.e. possible pairs) into subsets in terms of the types of the two entities, and for each subset to determine the most common class and assign this as the default to all instances in the subset. If the most common class is null, then all the entity pairs will be treated as unrelated.

More complicated baseline methods might use further criteria for subdividing the possible entity pairs into subsets for which most common classes are computed. In this paper, we also consider baselines using the left-right order of the two entities or whether they appear in the same sentence or not. Going too far along this route, however, can lead to more complicated methods that do not obviously deserve the title 'baseline', and can involve the work that is most naturally done by machine learning methods being laboriously reproduced as a manual feature engineering task.

## Results and discussion

### Feature selection

We next report experiments regarding the features most useful for relation extraction, using the features sets described in Table 3. We divide the discussion between the case of features sets that do not use syntactic parse information and those that do.

#### Non-syntactic features

The first group of experiments reported looks at the performance of relation extraction with non-parse feature sets. We followed an additive strategy for feature selection: starting with basic features, we added further features one set at a time. We measured the performance of the resulting classifier each time we added a new feature set. Results are shown in Table 4. The initial classifier used a tok6+atype feature set. Addition of both dir and dist features give significant improvements in all metrics, of around 10% *F*1 overall, in each case. This suggests that the linear text order of arguments, and whether relations are intra-or inter-sentential is important to classification. Addition of the str features also give good improvement in most metrics, again 10% *F*1 overall. Addition of part-of-speech information, in the form of pos features, however, leads to a drop in some metrics, overall *F*1 dropping by 1%. Unexpectedly, POS seems to provide little extra information above that in the surface string. Errors in POS tagging cannot be dismissed, and could be the cause of this. The existence of intervening entities, as coded in feature set inter, provides a small benefit. The inclusion of information about events, in the event feature set, is less clear-cut.

**Table 4:** Performance by feature set, non-syntactic features.

**Relation**	**Metric**	**tok6+ atype**	**+dir**	**+dist**	**+str**	**+pos**	**+inter**	**+event**	**allgen**	**notok**
**has_finding**	**P**	44	49	58	63	62	64	65	63	63
	**R**	39	63	78	80	80	81	81	82	82
	**F1**	39	54	66	70	69	71	72	71	71

**has_indication**	**P**	37	23	38	42	40	41	42	37	44
	**R**	14	14	46	44	44	47	47	45	47
	**F1**	18	16	39	39	38	41	42	38	41

**has_location**	**P**	36	36	50	68	71	72	72	73	73
	**R**	28	28	74	79	79	81	81	83	83
	**F1**	30	30	58	72	74	76	75	77	76

**has_target**	**P**	9	9	32	63	57	60	62	60	59
	**R**	11	11	51	68	67	67	66	68	68
	**F1**	9	9	38	64	60	63	63	63	62

**laterality_modifies**	**P**	21	38	73	84	83	84	84	86	86
	**R**	9	55	82	89	86	88	88	87	89
	**F1**	12	44	76	85	83	84	84	84	85

**negation_modifies**	**P**	19	54	85	81	80	79	79	77	81
	**R**	12	82	97	98	93	92	93	93	93
	**F1**	13	63	89	88	85	84	85	83	85

**sub_location_modifies**	**P**	2	2	55	88	86	86	88	88	87
	**R**	1	1	62	94	92	95	95	95	95
	**F1**	1	1	56	90	86	89	91	91	90

**Overall**	**P**	33	38	50	63	62	64	65	64	64
	**R**	22	36	70	74	73	75	75	76	76
	**F1**	26	37	58	68	67	69	69	69	70

Variation in performance by feature set, non-syntactic features. Features sets are abbreviated as in Table 3. For the first seven columns, features were added cumulatively to each other. The next two columns, allgen and notok, are as described in Table 3.

We were interested to see if generalising features could improve performance, as this had benefited our previous work in entity extraction. We replaced all surface string features with their root form, and POS features with their generalised POS form. This gave the results shown in column allgen. Results are not clear cut, in some cases better and in some worse than the previous best. Overall, there is no difference in *F*1. There is a slight increase in overall recall, and a corresponding drop in precision – as might be expected.

Both the tokN, and the str and pos feature sets provide surface string and POS information about tokens surrounding and between related entities. The former gives features from a window around each argument. The latter two provide more positional information. Do these two provide enough information on their own, without the windowed features? To test this, we removed the tokN features from the full cumulative feature set, corresponding to column +event of Table 4. The results, in column notok, show no clear change in performance, with some relationships improving, and some worsening. Overall, there is a 1% improvement in *F*1.

It appears that the bulk of performance is attained through entity type and distance features, with some contribution from positional surface string information. Performance is between 1% and 9% lower than CIAA for each relationship, with a best overall *F*1 of 70%, compared to a CIAA of 75%.

#### Syntactic features

The remaining feature selection experiments look at the impact of using features derived from a dependency parse analysis of the clinical texts made using the Stanford parser [39], which is a dependency parser that has been developed principally in relation to newswire texts. Despite the very different genre of our clinical texts, which are heavily laden with medical language, we did not attempt to adapt the Stanford parser to the domain, hoping rather that we could still benefit from exploiting whatever dependency analysis the parser is able to produce.

Table 5 reiterates the +event column of Table 4, corresponding to the accumulation of *all *non-syntactic feature sets, and gives results for augmenting this set with the syntactic features of dep and then also syndist. The syntactic features contribute mainly to finding the has_indication and negation_modifier relations, with an improved *F*1 of around 4% for each, while retaining performance for other relations. Overall we see a 3% increase in *F*1 to 72%, a step closer to the CIAA of 75%. The results illustrate that the SVM classifiers can exploit the more abstract information of underlying dependency relations, to generalise beyond the surface information of token strings and distances.

**Table 5:** Performance by feature set, syntactic features.

**Relation**	**Metric**	**+event**	**+dep**	**+syndist**
**has_finding**	**P**	65	73	74
	**R**	81	77	77
	**F1**	72	71	74

**has_indication**	**P**	42	42	43
	**R**	47	37	37
	**F1**	42	38	39

**has_location**	**P**	72	74	73
	**R**	81	86	86
	**F1**	75	79	78

**has_target**	**P**	62	65	71
	**R**	66	63	66
	**F1**	63	62	64

**laterality_modifies**	**P**	84	89	89
	**R**	88	84	90
	**F1**	84	85	89

**negation_modifies**	**P**	79	85	85
	**R**	93	97	93
	**F1**	85	90	88

**sub_location_modifies**	**P**	88	90	93
	**R**	95	95	95
	**F1**	91	92	94

**Overall**	**P**	65	71	71
	**R**	75	74	74
	**F1**	69	72	72

Variation in performance by feature set, syntactic features. The first column shows the cumulative +event system from Table 4. The next two columns show the effect of cumulatively adding syntactic features to this system. Syntactic features are as described in Table 3.

Given that the dependency analyses produced by the parser do not cross sentence boundaries (i.e. they are analyses of individual sentences), and since our syntactically-derived features are only computed for entities in the same sentence, we can expect their use to have a positive impact only on the discovery of *intra*-sentential relations. We found that a system using the syntactic feature set +syndist and applied to only the intra-sentential relations achieves an *F*1 of 77% (with *P *= 70%, *R *= 84%), as compared to a system using the non-syntactic feature set +event on the same intra-sentential subset of relations (corresponding to the *n <*1 column of Table 6), i.e. giving a 2% improvement in *F*1 overall.

**Table 6:** Performance by sentences.

		**Number of sentence boundaries between arguments**						
		
		**inter-**	**intra-**	**inter- and intra-sentential**				
**Relation**	**Metric**	**1 **≤ **n **≤ **5**	**n ***<***1**	**n **≤ **1**	**n **≤ **2**	**n **≤ **3**	**n **≤ **4**	**n **≤ **5**

**has_finding**	**P**	24	68	65	62	60	61	61
	**R**	18	89	81	79	78	78	77
	**F1**	18	76	72	69	67	68	67

**has_indication**	**P**	18	49	42	42	36	32	30
	**R**	17	59	47	42	42	39	38
	**F1**	16	51	42	39	37	34	33

**has_location**	**P**	n/a	74	72	73	72	72	72
	**R**	n/a	83	81	81	81	82	82
	**F1**	n/a	77	75	76	75	76	76

**has_target**	**P**	3	64	62	59	60	59	58
	**R**	1	75	66	64	62	61	61
	**F1**	2	68	63	61	60	60	59

**laterality_modifies**	**P**	n/a	86	84	86	86	86	87
	**R**	n/a	89	88	88	88	87	88
	**F1**	n/a	85	84	85	86	85	86

**negation_modifies**	**P**	n/a	80	79	79	80	80	80
	**R**	n/a	94	93	91	93	93	93
	**F1**	n/a	86	85	84	85	86	85

**sub_location_modifies**	**P**	n/a	89	88	88	89	89	89
	**R**	n/a	95	95	95	95	95	95
	**F1**	n/a	91	91	91	91	91	91

**Overall**	**P**	22	69	65	64	62	61	60
	**R**	17	83	75	73	71	70	70
	**F1**	19	75	69	68	66	65	65

Variation in performance, by number of sentence boundaries (*n*) crossed by a relationship. For all cases, the cumulative feature set +event of Table 4 was used. For the inter-sentential-only classifier 1 ≤ *n *≤ 5, the score fields for some relations are marked as n/a (not applicable). This is because some relations are either absent from the inter-sentential data (i.e. only ever appear intra-sententially), or are so rare that they do not appear in all training/test folds, and so a macro-average cannot be computed across the folds.

### Sentences spanned

Table 2 shows that although intra-sentential relations account for a clear majority (77%) of relationships, 23% are inter-sentential, with 10% of all relationships holding between entities in adjacent sentences. If we consider a relationship to cross *n *sentence boundaries, then the classifiers described above have mostly been trained on relationships crossing *n *≤ 1 sentence boundaries, i.e. with arguments in the same or adjacent sentences. What effect does including more distant relationships have on performance? To investigate this question, we trained classifiers for the subset of relationships found under a number of different distance conditions, in all cases using the cumulative feature set +event from Table 4, producing the results shown in Table 6. The first column shows results for a classifier of purely *inter*-sentential relations, for the case 1 ≤ *n *≤ 5 (which covers 85% of all inter-sentential relations), which can be seen to perform badly for the relations for which the approach applies. (Note that some relations occur across sentence boundaries either rarely or not at all, and so have been discounted in the results.) The next two columns compare classifiers trained on only intra-sentential relationships (*n *< 1) and those spanning up to one boundary (*n *≥ 1). The latter shows that even inclusion of relationships in adjacent sentences produces a 6% drop in overall *F*1 as compared to the purely intra-sentential case. Performance continues to drop as more inter-sentential relationships are included, as the remaining columns show.

A preliminary analysis of the data suggests that the further apart the related entities are, the more likely that clinical knowledge is required to extract the relationship, and such knowledge is clearly not available to the extraction approach described.

### Size of training corpus

The provision of sufficient training data for supervised learning algorithms is a limitation on their use. We examined the effect of training corpus size on relationship extraction. We selected subsets consisting of 25 and 50 documents from the C77 corpus, itself comprising 77 narratives, to produce sub-corpora that we refer to as C25 and C50, respectively. We trained two classifiers on these new corpora, again using the cumulative feature set +event, to give the results shown in Table 7. The table also shows the counts of the training instances for each relation type in the different corpora. Overall, performance improves as training corpus size increases (*F*1 rising from 63% to 69%), as expected. It is notable, however, that the performance for some relations (negation_modifies and has_location) appears to have plateaued even with this limited amount of training data, although it remains possible that a further increase in size may improve performance.

**Table 7:** Performance by corpus size.

		**Corpus size**		
		
**Relation**	**Metric**	**C25**	**C50**	**C77**
**has_finding**	**Count**	91	216	311
	**P**	66	63	65
	**R**	74	74	81
	**F1**	67	67	72

**has_indication**	**Count**	91	117	224
	**P**	22	25	42
	**R**	30	31	47
	**F1**	23	25	42

**has_location**	**Count**	127	199	364
	**P**	72	71	72
	**R**	76	80	81
	**F1**	73	74	75

**has_target**	**Count**	51	90	136
	**P**	65	49	62
	**R**	60	65	66
	**F1**	59	54	63

**laterality_modifies**	**Count**	57	73	128
	**P**	77	78	84
	**R**	69	68	88
	**F1**	72	69	84

**negation_modifies**	**Count**	34	67	101
	**P**	78	79	79
	**R**	80	93	93
	**F1**	78	84	85

**sub_location_modifies**	**Count**	30	43	76
	**P**	64	91	88
	**R**	64	85	95
	**F1**	64	86	91

**Overall**	**Count**	481	805	1340
	**P**	62	63	65
	**R**	65	71	75
	**F1**	63	66	69

Variation in performance by training corpus size. The "Count" row gives the number of training instances of a relation type, for the given corpus. The cumulative feature set +event of Table 4 was used.

### Extracting relations over extracted entities

The experiments described so far assume perfect entity recognition, using the entities of the gold standard as input to the relation extraction process, both for training and testing. This move is useful in allowing us to isolate the complexities of relation extraction from the vagaries of imperfect entity recognition when the method for performing the former task is under development. In operational use of the IE system, however, the limitations of entity recognition will impact the performance of relation extraction. To get a measure of this effect, we evaluated the system when applied to test data containing imperfect, extracted entities. The entity recognition approach is as described in [2], using a combination of lexical lookup and supervised ML. Lexical lookup uses the Termino terminology resource [40]. A Termino database is loaded with terms from the UMLS Metathesaurus [33]. Finite state recognisers are compiled from this database, and used to annotate terms in texts. These terms, together with a number of token-level features, are then used to train SVM classifiers: one for each entity type. This approach has been evaluated using ten fold cross validation over the C77 corpus (described above), achieving an overall *F*1 for entity recognition of 71%, macro-averaged across folds (full results are given in [2]).

We again used ten-fold cross validation to evaluate relation extraction with extracted entities. For each of the ten testing folds, the corresponding nine folds of gold standard data were used to train both an entity recognition model and a relation recognition model, the latter again using the +event feature set. The entity recognition model was then applied to the test fold to produce a version containing the recognised entities, and the relation recognition model applied to this version, i.e. using the recognised entities as the basis for creating the set of possibly-related entity pairs, to which the relation classifiers are applied. The relation results are then scored against the gold standard version of the test fold, with overall scores being macro-averaged across folds, as reported in Table 8. As anticipated, precision for relation recognition over extracted entities generally matches that over gold standard entities, but recall of relations suffers badly, with the overall *F*1 dropping from 70% to 48%. Performance does, however, remain close to IAA (Table 9), which measures an analogous human task in which annotators must find both entities and relations. Clearly, good relation extraction depends on good entity recognition.

**Table 8:** Performance over extracted entities.

**Relation**	**Metric**	**gold standard entities**	**extracted entities**
**has_finding**	**P**	63	62
	**R**	82	32
	**F1**	71	41

**has_indication**	**P**	44	44
	**R**	47	27
	**F1**	41	32

**has_location**	**P**	73	68
	**R**	83	49
	**F1**	76	55

**has_target**	**P**	59	47
	**R**	68	39
	**F1**	62	41

**laterality_modifies**	**P**	86	83
	**R**	89	76
	**F1**	85	74

**negation_modifies**	**P**	81	81
	**R**	93	53
	**F1**	85	60

**sub_location_modifies**	**P**	87	71
	**R**	95	24
	**F1**	90	31

**Overall**	**P**	64	63
	**R**	76	40
	**F1**	70	48

Performance of relation extraction over automatically extracted entities, compared to relation extraction using perfect gold standard entities. For relation extraction, the cumulative feature set +event of Table 4 was used.

**Table 9:** Overall performance evaluation.

**Relation**	**Metric**	**notok **(best system: non-syntactic)	+**syndist **(best system: syntactic)	**baseline**	**IAA**	**CIAA**
**has_finding**	**P**	63	74	65		
	**R**	82	77	76		
	**F1**	71	74	70	46	80

**has_indication**	**P**	44	43	0		
	**R**	47	37	0		
	**F1**	41	39	0	26	50

**has_location**	**P**	73	73	0		
	**R**	83	86	0		
	**F1**	76	78	0	55	80

**has_target**	**P**	59	71	0		
	**R**	68	66	0		
	**F1**	62	64	0	42	63

**laterality_modifies**	**P**	86	89	60		
	**R**	89	90	91		
	**F1**	85	89	72	73	94

**negation_modifies**	**P**	81	85	81		
	**R**	93	93	98		
	**F1**	85	88	88	66	93

**sub_location_modifies**	**P**	87	93	50		
	**R**	95	95	68		
	**F1**	90	94	58	49	96

**Overall**	**P**	64	71	36		
	**R**	76	74	48		
	**F1**	70	72	41	47	75

System best performance figures (from Tables 4 and 5), and comparison to baseline performance and to inter-annotator agreement scores.

The relation models used in this evaluation were trained over texts containing gold standard entities. For relation extraction over test data containing imperfect recognised entities, however, it may be that better performance would result with models also *trained *over data containing imperfect entities, but this issue can only be answered empirically.

### Summary of key results

Table 9 provides a summary of the key performance figures for the overall system, showing results for the best system configuration using only non-syntactic features (notok) and for the best one using syntactic features (+syndist). For most relation types, the syntactic system outperforms the non-syntactic one, with a macro-averaged *F*1 that is higher by 2–4%, (the exception being a 2% drop for the has_indication relation), giving a 2% increase in *F*1 overall. The table also provides scores for a baseline approach (to be detailed shortly) and for inter-annotator agreement, in both IAA and CIAA variants. We can see that IAA scores fall well below the system scores for all relation types, with an overall IAA of 47% compared to the overall system best of 72%, which shows simple IAA to be too pessimistic as an indicator of the likely upper bound of system performance, as expected. In contrast, CIAA scores are fairly close to, and mostly above, the system scores (the sole exception being a +syndist system score for has_target that is 1% above CIAA).

The baseline scores in the table are for a baseline system assigning different default relations to possibly-related entity pairs based on the types of the two entities, plus their left-right order and whether they appear in the same sentence or not. Other baselines were tried where only one of the latter two criteria, or neither, was used, but these showed much worse performance. The baseline scores were produced directly over the gold standard, i.e. with the set of possibly-related entity pairs being computed from the gold standard entities. For some relation types (e.g. has_target), we see *F*1 scores of 0%, showing that no correct instances of the relation were assigned. For some other relation types, however, this baseline approach works quite well, e.g. for has_finding we get a baseline *F*1 of 70%, which compares to a best system performance of 74% and a CIAA of 80%, whilst for negation_modifies we get a baseline *F*1 of 88%, which equals the best system performance and falls not far below the CIAA of 93%. Overall, however, the baseline method performs much worse than the best system, giving a macro-averaged *F*1 of 41% against a best system *F*1 of 72% and a CIAA of 75%. The simplest baseline, using only the types of the two entities, was found to score 0% for *all *measures (which followed from it having a null default for all cases).

## Conclusion

We have shown that it is possible to extract clinical relationships from text, using a supervised machine learning approach. IAA scores suggest that the task is difficult, but our system performs well, achieving an overall *F*1 of 72%, just 3% below corrected IAA. Although reasonable performance is achieved using quite simple surface/token-based features, our experiments indicate a real gain from using also features based on the more complex linguistic analysis provided by a dependency parser. We believe that this work has implications for clinical text mining more generally, given the success of our approach and its adaptability for other clinical domains, though further work to confirm our encouraging results should be carried out on a larger sample of narratives and relationship types. The technology used has proved scalable. The full CLEF IE system, including automatic entity recognition, is able to process a document in sub-second time on a commodity workstation. We have used the system to extract 6 million relations from over half a million patient documents, for use in downstream CLEF applications.

## Availability

The software described is open source and can be downloaded as part of GATE [41], except for the entity pairing component, which will be released shortly. We are currently preparing a UK research ethics committee application, for permission to release our annotated corpus.

## Competing interests

The authors declare that they have no competing interests.

## Authors' contributions

AR designed and built the system using the GATE framework, wrote most of the new components, prepared the data, contributed to evaluation tests, and drafted the manuscript. RG and MH are the principal and co-investigators of the project. They participated fully in the conceptual design of the CLEF IE approach and system, and of the reported experimental work, and contributed to the writing of the manuscript. YG implemented and evaluated the system augmentations for using syntactic features, and drafted the relevant manuscript sections. All authors read and approved the final manuscript.

## References

[B1] Rector A, Rogers J, Taweel A, Ingram D, Kalra D, Milan J, Singleton P, Gaizauskas R, Hepple M, Scott D, Power R (2003). CLEF – joining up healthcare with clinical and post-genomic research. Proceedings of UK e-Science All Hands Meeting 2003, Nottingham, UK.

[B2] Roberts A, Gaizauskas R, Hepple M, Guo Y (2008). Combining terminology resources and statistical methods for entity recognition: an evaluation. Proceedings of the Sixth International Conference on Language Resources and Evaluation, LREC 2008, Marrakech, Morocco.

[B3] Roberts A, Gaizauskas R, Hepple M (2008). Extracting Clinical Relationships from Patient Narratives. Proceedings of the Workshop on Current Trends in Biomedical Natural Language Processing.

[B4] Defense Advanced Research Projects Agency (1998). Proceedings of the Seventh Message Understanding Conference (MUC-7).

[B5] Chinchor N (1998). Overview of MUC-7/MET-2. Proceedings of the Seventh Message Understanding Conference (MUC-7), Fairfax, VA, USA.

[B6] Miller S, Crystal M, Fox H, Ramshaw L, Schwartz R, Stone R, Weischedel R (1998). Algorithms that learn to extract information: BBN: Description of the SIFT system as used for MUC-7. Proceedings of the Seventh Message Understanding Conference (MUC-7), Fairfax, VA, USA.

[B7] Doddington G, Mitchell A, Przybocki M, Ramshaw L, Strassel S, Weischedel R (2004). The Automatic Content Extraction (ACE) Program – Tasks, Data, & Evaluation. Proceedings of the 4th International Conference on Language Resources and Evaluation (LREC 2004), Lisbon, Portugal.

[B8] Nédellec C (2005). Learning language in logic – genic interaction extraction challenge. Proceedings of the ICML05 workshop: Learning Language in Logic (LLL05), Bonn, Germany.

[B9] BioCreAtIvE II – Protein-Protein Interaction Task. http://biocreative.sourceforge.net/biocreative_2_ppi.html.

[B10] Sager N, Lyman M, Bucknall C, Nhan N, Tick L (1994). Natural language processing and the representation of clinical data. J Am Med Inform Assoc.

[B11] Friedman C, Alderson P, Austin J, Cimino J, Johnson S (1994). A General Natural-language Text Processor for Clinical Radiology. J Am Med Inform Assoc.

[B12] Lussier Y, Borlawsky T, Rappaport D, Liu Y, Friedman C (2006). PhenoGO: assigning phenotypic context to gene ontology annotations with natural language processing. Pac Symp Biocomput.

[B13] Hahn U, Romacker M, Schulz S (2002). medsynDikate – a natural language system for the extraction of medical information from findings reports. Int J Med Inform.

[B14] Zweigenbaum P, Bachimont B, Bouaud J, Charlet J, Boisvieux JF (1995). A multi-lingual architecture for building a normalised conceptual representation from medical language. Proc Annu Symp Comput Appl Med Care.

[B15] Pakhomov S, Buntrock J, Duffy P (2005). High throughput modularized NLP system for clinical text. Proceedings of the 43rd Annual Meeting of the Association for Computational Linguistics, interactive poster and demonstration sessions, Ann Arbor, MI, USA.

[B16] Blaschke C, Andrade MA, Ouzonis C, Valencia A (1999). Automatic extraction of biological information from scientific text: protein-protein interactions. Proc Int Conf Intell Syst Mol Biol.

[B17] Thomas J, Milward D, Ouzounis C, Pulman S, Carroll M (2000). Automatic Extraction of Protein Interactions from Scientific Abstracts. Pac Symp Biocomput.

[B18] Pustejovsky J, Castano J, Zhang J, Kotecki M, Cochran B (2002). Robust Relational Parsing Over Biomedical Literature: Extracting Inhibit Relations. Pac Symp Biocomput.

[B19] Fundel K, Küffner R, Zimmer R (2007). RelEx-relation extraction using dependency parse trees. Bioinformatics.

[B20] Gaizauskas R, Demetriou G, Artymiuk P, Willett P (2003). Protein Structures and Information Extraction from Biological Texts: The PASTA System. Bioinformatics.

[B21] Rindflesch TC, Libbus B, Hristovski D, Aronson A, Kilicoglu H (2003). Semantic relations asserting the etiology of genetic diseases. AMIA Annu Symp Proc.

[B22] Ahlers CB, Fiszman M, Demner-Fushman D, Lang FM, Rindflesch TC (2007). Extracting Semantic Predications from Medline Citations for Pharmacogenomics. Pac Symp Biocomput.

[B23] Grover C, Haddow B, Klein E, Matthews M, Nielsen L, Tobin R, Wang X (2007). Adapting a Relation Extraction Pipeline for the BioCreAtIvE II Task. Proceedings of the BioCreAtIvE II Workshop Madrid, Spain.

[B24] Bundschus M, Dejori M, Stetter M, Tresp V, Kriegel HP (2008). Extraction of semantic biomedical relations from text using conditional random fields. BMC Bioinformatics.

[B25] Bunescu R, Ge R, Kate R, Marcotte E, Mooney R, Ramani A, Wong Y (2005). Comparative experiments on learning information extractors for proteins and their interactions. Artif Intell Med.

[B26] Goadrich M, Oliphant L, Shavlik J (2005). Learning to Extract Genic Interactions Using Gleaner. Proceedings of the ICML05 workshop: Learning Language in Logic (LLL05), Bonn, Germany.

[B27] Giuliano C, Lavelli A, Romano L (2006). Exploiting Shallow Linguistic Information for Relation Extraction from Biomedical Literature. Proceedings of the 11th Conference of the European Chapter of the Association for Computational Linguistics (EACL06), Trento, Italy.

[B28] Zelenko D, Aone C, Richardella A (2002). Kernel Methods for Relation Extraction. Proceedings of the Conference on Empirical Methods in Natural Language Processing (EMNLP), Philadelphia, PA, USA.

[B29] Zhou G, Su J, Zhang J, Zhang M (2005). Exploring Various Knowledge in Relation Extraction. Proceedings of the 43rd Annual Meeting of the Association for Computational Linguistics, Ann Arbor, MI, USA.

[B30] Bunescu RC, Mooney RJ (2005). A Shortest Path Dependency Kernel for Relation Extraction. Proceedings of the Human Language Technology Conference and Conference on Empirical Methods in Natural Language Processing.

[B31] Katrenko S, Adriaans P (2007). Learning Relations from Biomedical Corpora Using Dependency Trees. Knowledge Discovery and Emergent Complexity in Bioinformatics.

[B32] Chapman W, Bridewell W, Hanbury P, Cooper G, Buchanan B (2001). A simple algorithm for identifying negated findings and diseases in discharge summaries. J Biomed Inform.

[B33] Lindberg D, Humphreys B, McCray A (1993). The Unified Medical Language System. Methods Inf Med.

[B34] Roberts A, Gaizauskas R, Hepple M, Demetriou G, Guo Y, Setzer A, Roberts I (2008). Semantic Annotation of Clinical Text: The CLEF Corpus. Proceedings of Building and evaluating resources for biomedical text mining: workshop at LREC 2008, Marrakech, Morocco.

[B35] Cunningham H, Maynard D, Bontcheva K, Tablan V (2002). GATE: A Framework and Graphical Development Environment for Robust NLP Tools and Applications. Proceedings of the 40th Anniversary Meeting of the Association for Computational Linguistics, Philadelphia, PA, USA.

[B36] Rindflesch T, Fiszman M (2003). The interaction of domain knowledge and linguistic structure in natural language processing: interpreting hypernymic propositions in biomedical text. J Biomed Inform.

[B37] Li Y, Bontcheva K, Cunningham H (2005). SVM Based Learning System for Information Extraction. Deterministic and statistical methods in machine learning: first international workshop.

[B38] Wang T, Li Y, Bontcheva K, Cunningham H, Wang J (2006). Automatic Extraction of Hierarchical Relations from Text. The Semantic Web: Research and Applications 3rd European Semantic Web Conference, ESWC 2006.

[B39] Klein D, Manning CD (2003). Accurate unlexicalized parsing. Proceedings of the 41st Annual Meeting on Association for Computational Linguistics.

[B40] Harkema H, Gaizauskas R, Hepple M, Roberts A, Roberts I, Davis N, Guo Y, Hirschman L, Pustejovsky J (2004). A Large Scale Terminology Resource for Biomedical Text Processing. HLT-NAACL 2004 Workshop: BioLINK Linking Biological Literature, Ontologies and Databases.

[B41] GATE – General Architecture for Text Engineering. http://gate.ac.uk.

